# Impact of remote vital sign monitoring on health outcomes in acute respiratory infection and exacerbation of chronic respiratory conditions: systematic review and meta-analysis

**DOI:** 10.1183/23120541.00393-2022

**Published:** 2023-04-24

**Authors:** Samuel Thomas Creavin, Megha Garg, Alastair D. Hay

**Affiliations:** Centre for Academic Primary Care, Population Health Sciences, Bristol Medical School, Bristol, UK

## Abstract

**Background:**

Our aim was to investigate the effectiveness of virtual wards on health outcomes in patients with acute respiratory infection.

**Methods:**

We searched four electronic databases from January 2000 to March 2021 for randomised controlled trials (RCTs). We included studies in people with acute respiratory illness or an acute exacerbation of a chronic respiratory illness, where a patient or carer measured vital signs (oximetry, blood pressure, pulse) for initial diagnosis and/or asynchronous monitoring, in a person living in private housing or a care home. We performed random-effects meta-analysis for mortality.

**Results:**

We reviewed 5834 abstracts and 107 full texts. Nine RCTs were judged to be relevant for inclusion, in which sample sizes ranged from 37 to 389 (total n=1627) and mean ages ranged between 61 and 77 years. Five were judged to be at low risk of bias. Five RCTs had fewer hospital admissions in the intervention (monitoring) group, out of which two studies reported a significant difference. Two studies reported more admissions in the intervention group, with one reporting a significant difference. We were unable to perform a meta-analysis on healthcare utilisation and hospitalisation data due to lack of outcome definition in the primary studies and variable outcome measurements. We judged two studies to be at low risk of bias. The pooled summary risk ratio for mortality was 0.90 (95% CI 0.55–1.48).

**Conclusion:**

The limited literature for remote monitoring of vital signs in acute respiratory illness provides weak evidence that these interventions have a variable impact on hospitalisations and healthcare utilisation, and may reduce mortality.

## Abstract 2

**Remote monitoring may have a beneficial effect in acute respiratory illness or acute exacerbation of chronic respiratory disease to mitigate the crisis of capacity for many healthcare providers** https://bit.ly/3jgmVda

## Background

During the coronavirus disease 2019 (COVID-19) pandemic several healthcare providers developed innovative models of care for people who had a recent diagnosis of severe acute respiratory syndrome coronavirus 2 infection, but did not currently require hospitalisation [1]. The intention of these models of care was to reduce the number of people who required hospitalisation to monitor their condition for deterioration, and to mitigate the crisis of capacity for many healthcare providers [2].

A particular clinical challenge of COVID-19 disease is the phenomena of profoundly low oximetry without dyspnoea, so called “silent hypoxia” [3, 4], and UK guidelines have recommended assessment of pulse oximetry in people with suspected COVID-19 (including those who are breathless, at high risk or ill) [5]. However, the principle of remotely monitoring vital signs in people with acute respiratory illness has precedent in exacerbations of chronic disease, such as asthma and COPD and is potentially applicable to other cardiorespiratory illnesses such as pneumonia or acute pulmonary oedema [1].

Other authors have reviewed and evaluated some of the innovative models of care for remote monitoring in COVID-19 [6]. Models for monitoring included online platforms, paper forms, telephone calls or wearable sensors, and training for patients/carers was key to the success of the intervention [6]. The potential for using innovative models of care for other clinical conditions is recognised and unanswered questions have been identified, which include “what is the impact of remote home monitoring on patient outcomes?” [1].

Our objective was to systematically review the randomised controlled trials (RCTs) relating to telemonitoring in acute respiratory illness. We undertook a systemic review to answer the question “in people with acute respiratory illness, or acute exacerbation of chronic respiratory disease, what is the impact of remote baseline evaluation and/or repeated measurement of self-assessed vital signs including blood pressure, pulse rate and oximetry, using standard assessment tools such as sphygmomanometer and pulse oximeter, compared to no measurement or one-off measurement in a healthcare setting, on healthcare utilisation, and patient relevant outcomes such as mortality, following initial consultation?”.

## Methods

We published a protocol for our systemic review on PROSPERO (www.crd.york.ac.uk/prospero identifier CRD42021241094). We searched Ovid MEDLINE, Ovid Embase, the Cumulated Index to Nursing and Allied Health Literature and medRxiv with no language restrictions from 2000 to 10 March 2021.

We included RCTs and excluded uncontrolled studies, nonrandomised studies and case series or uncontrolled cohorts, since these designs are at higher risk of bias. We used Covidence for data management (www.covidence.org).

### Participants

We included studies in people with an acute respiratory illness, including COVID-19, or an acute exacerbation of a chronic respiratory illness. We defined acute respiratory illness as a worsening of respiratory symptoms (cough, dyspnoea, sputum) over a short duration (up to 10 days), regardless of whether someone had a pre-existing chronic respiratory disease such as asthma or COPD. We included studies in a care home or nursing home, as long as someone who worked in that setting, or the patient, measured vital signs.

### Intervention and control

We included studies where a patient or carer measured vital signs (blood pressure, pulse rate, oximetry) using standard assessment tools such as sphygmomanometer and pulse oximeter, remotely to a healthcare setting, for initial diagnosis and/or asynchronous monitoring. We also included studies where a professional person measured vital signs in the person's normal place of residence. We excluded studies where the measurement of vital signs took place exclusively in healthcare settings such as general practices or hospitals, or where apps derived vital signs using novel technology, which is not in routine use in typical healthcare settings in the UK National Health Service. We included studies where there was a control group of either no measurement of vital signs, or a one-off measurement of vital signs in a healthcare setting.

### Setting

We included studies in the community (*i.e.* outside of hospitals) where patients were in their usual place of residence, including a care home or nursing home. We defined a hospital as a facility where medical staff (*i.e.* doctors) are typically available on site 24 h a day. We included studies conducted in community hospitals, where a doctor is nonresident, as we considered care in these settings to be more reflective of care in nursing or care homes than hospitals.

### Outcome

We selected studies that reported any data on healthcare utilisation. We hoped to identify studies reporting on length of stay or repeat measures of healthcare consultation in the 10 days following an initial evaluation (including out-of-hours), emergency department attendances, hospital admissions and readmission. We anticipated that the main measure of effect would be odds ratios for further healthcare utilisation in the following 10 days in the intervention and control group, but we included all studies that met the criteria for participants, intervention and setting to be inclusive. In addition, we aimed to collect data on any significant harms associated with remote evaluation or monitoring in acute respiratory illness and on patient experience. We anticipated that for our additional outcome, data were likely to be poorly quantified and so we extracted whatever measures were available.

### Data extraction (selection and coding)

One investigator screened the identified studies for relevance at the title and abstract stage to identify studies that required full-text review and a second author verified selection in a 10% random sample of titles/abstracts. A single author reviewed articles at the full-text stage and a second author reviewed the full text of a 10% random sample of full-text articles. At the full-text stage, reasons for exclusion were categorised as ineligible based on study setting, study design, population, condition, intervention and outcome.

For all included studies, one author extracted data, which a second author verified. Data extraction included details of the study population (demographics, age, sex, country), dates of data collection, illness, setting, recruitment to the study, randomisation (where appropriate), allocation concealment, blinding to group allocation, and outcomes. We piloted data extraction forms on two studies. We contacted authors of primary studies by email to attempt to obtain further details where necessary.

### Risk-of-bias (quality) assessment

We assessed risk of bias using the Cochrane Risk-of-Bias tool for RCTs (RoB 2) [7]. We planned to restrict our main analysis to studies that were not at high risk of bias in more than two domains, and to perform a sensitivity analysis including all studies. The certainty of individual outcomes was graded (as high, moderate, low, very low) using the modified Grading of Recommendations Assessment, Development and Evaluation (GRADE) approach (www.gradepro.org).

### Strategy for data synthesis

We summarised characteristics of each study in summary tables including details regarding the population, intervention, control and outcomes. We summarised data on healthcare utilisation using ratios or count, based on the availability of data.

### Analysis of subgroups or subsets

We planned to perform a subgroup analysis of the impact of the interventions in the following groups: children compared to adults; and adults living in a care facility with professional staff (including care homes, nursing homes, community hospitals) and adults living in their own home.

Limitations of data reporting in the included studies prevented us from undertaking any subgroup analysis. We classified studies by the acuity of illness in the recruited participants, using two groups of studies: “possibly stable” that appeared to recruit people who were still recovering from an acute respiratory illness; and “unstable” that recruited people in the acute phase of a respiratory illness.

### Meta-analysis

We performed a Mantel–Haenszel (M–H) random-effects meta-analysis using Review Manager version 5.4.1 to summarise the effect of the intervention on the risk of mortality (expressed as risk ratio (95% CI)) in the studies that we judged to be at lowest risk of bias [8]. We used M–H method as both the M–H and inverse-variance method use the moment-based approach to estimate the amount of between-studies variation. However, the difference between the two methods is subtle: the former estimates the between-study variation by comparing each study's result with a M–H fixed-effect meta-analysis result, whereas the latter estimates it by comparing each study's result with an inverse-variance fixed-effect meta-analysis result. In practice, the difference is likely to be trivial [9]. We used I^2^ statistics as a measure of heterogeneity. In addition, we performed a sensitivity analysis where we included all the studies that included data on this outcome.

## Results

We retrieved 6146 citations, and after we excluded duplicates there were 5834 titles and abstracts for review. We reviewed the full text of 107 articles and excluded 98 of these, most commonly because the study used an ineligible study design (62 studies) or because the study did not report on vital signs (15 studies). We included nine randomised controlled trials in our review (supplementary figure S1).

### Characteristics of included RCTs

Table 1 summarises the characteristics of the nine included RCTs. Two studies were conducted in Italy [12, 18], two in Denmark [14, 15] and one each in Hong Kong [16], Japan [17], Canada [11], USA [10] and the UK [13]. We classified three studies as including unstable patients [14, 15, 17] and six as possibly stable [10–13, 16, 18]. The sample size of included randomised trials ranged from 37 [17] to 389 [10]. The mean age of participants ranged from 61 years [18] to 77 years [17]. The proportion of male participants ranged from 39% in Jakobsen *et al.* [14] and Sorknaes *et al.* [15] to 98% in Chau *et al*. [16]. All trials measured oximetry using a finger pulse oximeter; six studies reported that they also measured heart rate (all except [10, 17, 18]); and Chatwin *et al.* [13] also measured blood pressure using a sphygmomanometer. Frequency of remote assessment ranged from thrice daily [16], daily [14, 15], weekdays [10, 12, 13] and once a week [11].

**TABLE 1:** Characteristics of included randomised controlled trials of remote monitoring in people with COPD

**First author, year [reference]**	**Country**	**Participants**										**Intervention**		
		**Recruited when^#^**	**Total number (data analysed)^¶^**			**Mean age years**			**Male**			***S*_pO_2__ monitoring**	**How other vital signs (blood pressure/heart rate) were monitored**	**Frequency of remote assessment**
			**Overall**	**Intervention**	**Control**	**Overall**	**Intervention**	**Control**	**Overall**	**Intervention**	**Control**			
**Koff, 2021 [**10**]**	USA	Possibly stable	389	249	140	68	68.3	68.4	238^+^ (61.2)	145^+^ (66.7)	93^+^ (58)	Finger pulse oximeter		Once daily Monday to Friday
**Kessler, 2018 [**11**]**	Canada	Possibly stable	319	157	162	66.9	67.3	66.6	222 (69.6)	109 (69.4)	113 (69.8)	Finger pulse oximeter	Heart rate: finger pulse oximeter	At least once a week
**Vianello, 2016 [**12**]**	Italy	Unstable/possibly stable	262	181	81	76.1^+^	75.9	76.4	188 (71.8)	129 (71.3)	59 (73.1)	Finger pulse oximeter	Heart rate: finger pulse oximeter	Once daily Monday to Friday
**Chatwin, 2016 [**13**]**	UK	Possibly stable	61			61.8			29 (48)			Finger pulse oximeter	Heart rate: finger pulse oximeter Blood pressure: sphygmomanometer	Once daily Monday to Friday
**Jakobsen, 2015 [**14**]**	Denmark	Unstable	57	29	28	71^+^	71^+^	71^+^	22 (38.6)	11 (39.3)	11 (37.9)	Finger pulse oximeter	Heart rate: finger pulse oximeter	Daily
**Sorknaes, 2013 [**15**]**	Denmark	Unstable	242	121	121	72	71	72	94^+^ (38.8)	48^+^ (40)	46^+^ (38)	Finger pulse oximeter	Heart rate: finger pulse oximeter	Daily
**Chau, 2012 [**16**]**	Hong Kong	Possibly stable	40	22	18	72.9	73.5	72.2	39 (97.5)	21 (95.5)	18 (100)	Finger pulse oximeter	Heart rate: finger pulse oximeter	Thrice daily Monday to Friday
**Kamei, 2013 [**17**]**	Japan	Unstable	37	20	17	76.8^+^	76	77.7	36 (100)			Finger pulse oximeter		
**Vitacca, 2009 [**18**]**	Italy	Possibly stable	220	118	102	61.2^+^	61.2	61.1	149 (67.7)	75 (64)	74 (72)	Finger pulse oximeter		Unclear

Data are presented as n or n (%), unless otherwise stated. *S*_pO_2__: peripheral oxygen saturation. ^#^: possibly stable, still recovering from an acute respiratory illness; unstable, in the acute phase of a respiratory illness; ^¶^: includes participants as per intention-to-treat approach; therefore, studies have smaller numbers of total participants compared to table 2; ^+^: as calculated by authors M. Garg and S.T. Creavin.

### Risk of bias in included studies

**FIGURE 1:**
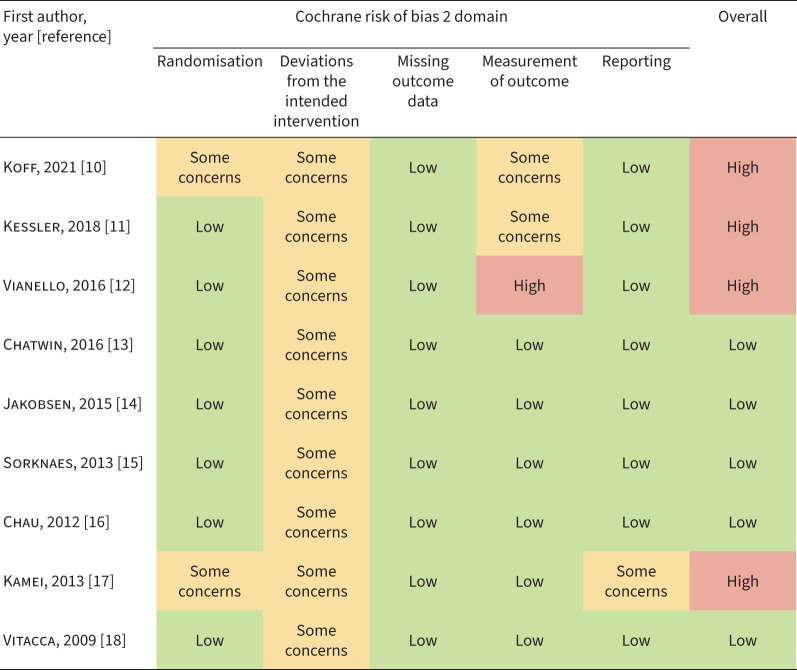
Author-judged risk of bias in included randomised controlled trials

Figure 1 shows the risk of bias in the included randomised trials. Overall, we determined that five of the nine RCTs were at low risk of bias, and the remaining four were at high risk of bias [10–12, 17]. Among the studies at high risk of bias, we had concerns about randomisation in two [10, 17], concerns about outcome measurement in three [10–12] and concerns about reporting in one [17]. We had some concerns about deviations from the intended intervention in all studies, because participants, carers and people delivering the interventions were aware of the participants’ group allocation in the trial. This affects the health-related behaviours between the participants of different intervention groups along with differences in implementation of intervention between groups. Moreover, the trialists did not report whether these deviations arose because of the trial context. We provide a rationale for our rating on risk of bias in supplementary table S1 using quoted text excerpts from the primary studies.

**FIGURE 2:**
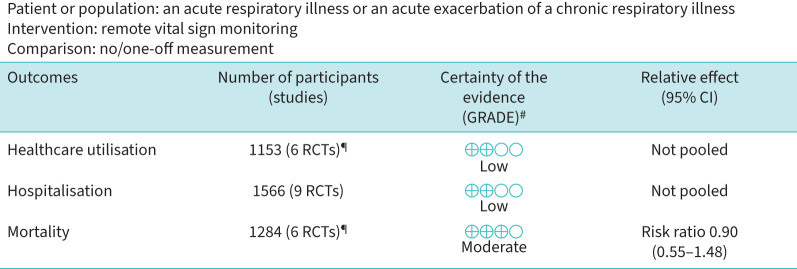
Grading of Recommendations Assessment, Development and Evaluation (GRADE) evaluation: remote monitoring for acute respiratory illness and exacerbations. The risk in the intervention group (and its 95% confidence interval) is based on the assumed risk in the comparison group and the relative effect of the intervention (and its 95% confidence interval). RCT: randomised controlled trial. ^#^: high certainty (we are very confident that the true effect lies close to that of the estimate of the effect), moderate certainty (we are moderately confident in the effect estimate: the true effect is likely to be close to the estimate of the effect, but there is a possibility that it is substantially different), low certainty (our confidence in the effect estimate is limited: the true effect may be substantially different from the estimate of the effect), very low certainty (we have very little confidence in the effect estimate: the true effect is likely to be substantially different from the estimate of effect); ^¶^: data for healthcare utilisation and hospitalisation were not pooled due to lack of outcome definition in the primary studies and variable outcome measurements; there were no measures of variability in the primary studies.

On GRADE evaluation, evidence for healthcare utilisation and hospitalisation outcome measures were rated as “low certainty”, whereas evidence for mortality was found to be moderately confident in the effect estimate. We downgraded the studies for all outcomes based on some concerns in their overall risk of bias. Studies were downgraded for healthcare utilisation and hospitalisation for heterogeneity (inconsistency) in outcome reporting (figure 2).

### Effect of interventions

Table 2 summarises the impact of remote monitoring on healthcare utilisation including emergency visits, office and hospital visits, general practitioner (GP) visits and home visits, and hospitalisation and death. Measures for healthcare utilisation differed between studies. Koff *et al.* [10], Vianello *et al.* [12] and Vitacca *et al.* [18] reported fewer emergency visits in the telemonitoring group compared to the usual-care group, whereas Chau *et al.* [16] reported higher incidence of emergency visits in the remote monitoring group (n=7) as compared to the control group (n=3). Koff *et al.* [10] reported a greater reduction in urgent office visits per patient for 12 months in the telemonitoring group (from 0.30 to −0.66) than the control group (from 0.26 to 0.10; p<0.0001). Vianello *et al.* [12] reported fewer office visits in the intervention group (incidence rate per year 1.41) than the control group (incidence rate per year 1.72). Chatwin *et al.* [13] found a statistically significantly higher number of home visits in the intervention group (mean at 6 months 4) than in the control group (mean at 6 months 0.75). Mean office visits (telemonitoring group 3.79, control group 3.23) and GP visits (telemonitoring group 5.75, control group 5.17) at 6 months were slightly increased in the telemonitoring group when compared to the control group. Urgent telephone calls were found to be statistically significantly lower in remotely monitored patients in the Vitacca *et al.* [18] study (urgent calls per month 0.07) than the usual-care group (urgent calls per month 0.22).

**TABLE 2:** Impact of remote monitoring on healthcare utilisation, hospitalisation and death

**First author, year [reference]**	**Healthcare utilisation**												**Reasons for possibly significant results as stated in the study**
	**Emergency visits**		**Office and hospital visits**		**GP visits (including urgent)**		**Home visits**		**Hospitalisation**		**Mortality (incidence in study duration)**		
	**Intervention**	**Control**	**Intervention**	**Control**	**Intervention**	**Control**	**Intervention**	**Control**	**Intervention**	**Control**	**Intervention**	**Control**	
**Koff, 2021 [**10**]**	Reduced 0.35 visits per patient for 12 months	Reduced 0.18 visits per patient for 12 months	Urgent visits were reduced by 0.66 visits per patient^#^ for 12 months	Urgent visits were increased by 0.10 visits per patient^#^ for 12 months					Hospitalisation reduced by 0.21 visits per patient for 12 months	Hospitalisation reduced by 0.11 visits per patient for 12 months	4/352	6/159	Coordinators called all patients with red flags in the intervention group and used discretion in contacting patients who had persistent red or yellow flags Coordinators helped resolve clinical problems directly or by calling the participant's primary care provider
**Kessler, 2018 [**11**]**									All-cause acute care ward days at 12 months =17.4 Incidence of all-cause hospitalisation at 12 months 157	All-cause acute care ward days at 12 months 22.6 Incidence of all cause hospitalisation at 12 months 160	3/157^#^	23/162^#^	It is possible that the intervention reduced mortality by successfully optimising the self-management of exacerbations, leading to early and prompt treatment, which could have prevented additional complications, including death. The home monitoring aspect of the intervention may have also contributed to the success of the intervention by providing a means of rapidly communicating symptoms and disease severity variables to case managers, which may have shortened the time from the beginning of an exacerbation to the institution of appropriate therapy
**Vianello, 2016 [**12**]**	Incidence rate per year 1.29	Incidence rate per year 1.37	Incidence rate per year 1.41	Incidence rate per year 1.72					Incidence rate per year: First admission due to AECOPD 0.74 Readmission due to AECOPD 0.07^#^	Incidence rate per year: First admission due to AECOPD 0.84 Readmission due to AECOPD 0.15^#^	23/181	9/81	The p-value here is 0.04 (quite arbitrary and results should be interpreted with extreme caution) First, the number of hospital admissions was very low even in the control group Second, variations in heart rate and *S*_pO_2__ cannot always reflect changes in patients’ health status, leading to underestimation and treatment delay of acute exacerbation episodes Third, our trial was not powered for the outcome of hospitalisation
**Chatwin, 2016 [**13**]**			Mean at 6 months 3.79	Mean at 6 months 3.23	Mean at 6 months 5.75	Mean at 6 months 5.17	Mean at 6 months 4^#^	Mean at 6 months 0.75^#^	Mean admissions at 6 months 0.63^#^	Mean admissions at 6 months 0.32^#^	Mean admissions at 6 months 0.63^#^	Mean admissions at 6 months 0.32^#^	Home visits were carried out in the intervention group if the patient was not responding to telephone advice The main triggers for a home visits were fall in *S*_pO_2__, accompanied by symptoms The criteria for hospital admission are dependent on local hospital policy as not all patients were directly admitted to our centre
**Jakobsen, 2015 [**14**]**									Number of readmissions in 180 days 1.08	Number of readmissions in 180 days 2.39	3/29	4/28	
**Sorknaes, 2013 [**15**]**					Number of consultations in 26 weeks 38				Mean number of AECOPD readmissions (and hospital days) at 4 weeks 0.36 (0.97) 8 weeks 0.63 (2.06) 12 weeks 0.77 (2.46) 26 weeks 1.22 (3.88)	Mean number of AECOPD readmissions (and hospital days) at 4 weeks 0.28 (1.1) 8 weeks 0.47 (2.13) 12 weeks 0.72 (3.25) 26 weeks 1.28 (5.16)			A possible explanation for the high readmission rate in the intervention group might be that the patients were readmitted at an earlier stage, so they were readmitted before they became seriously ill
**Chau, 2012 [**16**]**	Incidence at 2 months 7	Incidence at 2 months 3							Number of readmissions at 2 months 7 Average hospitalisation days at 2 months 2.16	Number of readmissions at 2 months 3 Average hospitalisation days at 2 months 0.78			This study was limited by the short duration of implementation and the small number of participants
**Kamei, 2013 [**17**]**									Number of readmissions at 3 months 4	Number of readmissions at 3 months 4	0/20	0/17	
**Vitacca, 2009 [**18**]**	Visits per month 0.07	Visits per month 0.10			Urgent calls per month 0.07^#^	Urgent calls per month 0.22^#^			Number of admissions per month 0.14^#^	Number of admissions per month 0.22^#^	21/118	23/120	In 60% of cases the nurse/tutor alone was able to resolve clinical or logistical problems A reduction in hospital admissions in the intervention group may be because algorithms on computers were used by nurses to follow patients after hospital discharge. This may have also been favoured by the prompt availability and use of the *S*_pO_2__ device, which has provided important data for staff decisions about diagnosis of hypoxaemia and oxygen and/or mechanical ventilation prescription

Data are presented as n/N, unless otherwise stated. GP: general practitioner; AECOPD: acute exacerbation of COPD; *S*_pO_2__: peripheral oxygen saturation. ^#^: statistically significant difference; all measures of variability that are available in primary studies are reported.

Regarding the role of remote monitoring in hospitalisation and death in the nine included RCTs, most studies were underpowered to detect differences in mortality. Kessler *et al.* [11] reported that participants in the intervention group had a statisically significant reduction in mortality compared to those in the control group (three out of 157 *versus* 23 out of 162). Koff *et al.* [10], Vianello *et al.* [12], Jakobsen *et al.* [14] and Vitacca *et al.* [18] reported reduction in mortality among participants enrolled in the telemonitoring group compared to usual care. Five studies [10–12, 14, 18] reported fewer hospitalisations in the intervention group, but the specific outcomes varied. For example, Kessler *et al.* [11] reported the intervention group had 17.4 days in acute care wards (total numbers of unplanned hospital admissions 157) in the study period, compared to 22.6 days in the control group (total numbers of unplanned hospital admissions 160), whereas Jakobsen *et al.* [14] reported that the number of readmissions in 180 days was 1.08 in the intervention group compared to 2.39 in the control group. Two studies reported a statistically significant reduction in hospitalisations in the intervention group: Vianello *et al.* [12] reported an incidence rate per year of 0.07 readmissions for acute exacerbations of COPD for all patients in the intervention group compared to 0.15 for all patients in the control group, and Vitacca *et al.* [18] reported 0.14 admissions per month for all patients in the intervention group compared to 0.22 admissions per month for all patients in the control group. In contrast, two studies reported more hospitalisations in the intervention group: Chatwin *et al.* [13] reported 0.63 mean admissions at 6 months in the intervention group compared to 0.32 in the control group, and Chau *et al.* [16] reported seven readmissions in the study period in the intervention group compared to three in the control group.

### Meta-analysis for effect of intervention on mortality

**FIGURE 3:**
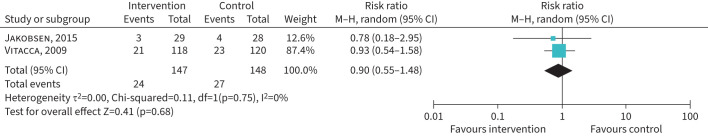
Meta-analysis for mortality among low risk-of-bias studies (effect size represented as risk ratio). M–H: Mantel–Haenszel.

Figure 3 shows the forest plot for the meta-analysis of the impact of the intervention on mortality, in the two studies [14, 18] that had data on this outcome and where we judged them to be at low risk of bias. In the random effects meta-analysis, the summary risk ratio was 0.90 (95% CI 0.55–1.48), I^2^ for heterogeneity 0%. In a sensitivity analysis where we included four further studies [10–12, 17] that had data on mortality but that we judged to be at high of bias, the summary risk ratio was 0.56 (95% CI 0.27–1.17), I^2^ for heterogeneity 68% (supplementary figure S2).

## Discussion

### Summary of main findings

We systematically reviewed the literature on the use of remote home monitoring for outcomes related to hospital admission and healthcare utilisation and found that the available data suggest that remote monitoring may reduce mortality and hospitalisations. There was a wide range of outcomes used by different studies, suggesting that there is uncertainty in what the most relevant outcomes are for patients, clinicians and commissioners. Most studies were in older people (mean age for most studies was >60 years) and in people with underlying respiratory disease. We found no studies in people with acute pulmonary oedema and no RCTs in COVID-19. Many studies were relatively small and potentially underpowered to draw firm conclusions about potential issues regarding safety or implementation of the intervention.

### Strengths and limitations

We conducted a thorough and systematic search which helped us retrieve two additional articles. We systematically assessed the risk of bias in the included studies, providing a rationale for our judgements, and verified the extracted data in duplicate. We did not limit the search to the English language.

There were differences between our protocol and the methods in the review. The ongoing COVID-19 pandemic impacted availability of clinicians involved in the study. Therefore, we did not have all titles and abstracts reviewed by a second author due to resource constraints caused by the ongoing pandemic. We did complete a 10% random verification of excluded articles and none of the excluded titles/abstracts were relevant for inclusion. There was no measure of variability reported for some outcome data in the primary studies, which means we cannot report this in figure 2 or table 2. We were unable to perform a meta-analysis on healthcare utilisation and hospitalisation data due to lack of outcome definition in the primary studies and variable outcome measurements, as studies assessed widely different clinical parameters at different follow-up durations, and the subsequent heterogeneity therefore prevented meta-analysis. This was marked using the GRADE evaluation, where we observed a very low certainty of evidence for healthcare utilisation and hospitalisation outcome measures.

### Comparison to the literature

A limited number of studies have reported healthcare data on the use of remote monitoring, in patients with acute respiratory illnesses, *via* standardised technologies. One systematic review was done of remote monitoring models for people with acute COVID-19 infection, but the nature of that review meant that the included studies were uncontrolled [6]. A second review systematically reviewed health-related outcomes for remote monitoring exclusively in people with COPD [19], whereas we extended our search to patients with all acute respiratory illnesses. We retrieved more abstracts than the previous review [19] (5834 abstracts *versus* 76 abstracts) and found a similar overall effect of interventions. We included a smaller number of studies in our review than the previous review [19], which we attribute to the strict inclusion criteria of the acute respiratory illness population. Other related systematic reviews synthesised a large number of studies (numbering between seven and 91 included studies) including either both acute and chronic COPD patients [20–24] or a wide range of diseases such as COVID-19, diabetes, hypertension and cardiovascular diseases [25, 26]. We did not include these studies in our review because they included patients with chronic and/or multiple illness (other than respiratory) and the data could not be segregated.

In a variety of diseases, including people who had stable chronic disease, Taylor *et al.* [26] studied the effectiveness of remote patient monitoring and included 91 citations in their review. Data from ∼50% of the included studies suggested reduced hospital admissions and length of stay; however, the other half reported no change. Two studies in the Taylor *et al.* [26] review reported a higher hospitalisation rate in the COPD telehealth group. In people with stable COPD, a Cochrane review by McLean *et al.* [24] synthesised data from 29 studies to investigate the effect of 10 telehealth interventions for people with COPD and concluded that telehealth reduced hospitalisation rate; however, they found scarce evidence for reduction in re-admissions. In people with stable chronic pulmonary and cardiac diseases, Polisena *et al.* [21] and Paré *et al.* [25] reported reduction in hospital admissions and average length of stay among remotely monitored patients. In people with stable chronic COPD, Ram *et al.* [22] (seven studies) and Janjua *et al.* [23] (29 studies) observed insignificant differences in readmission and hospitalisation rates, respectively.

Overall, the literature demonstrates a consistent trend that remote patient monitoring in acute illness may reduce mortality, but the impact on healthcare utilisation is more variable. However, we acknowledge that existing data are limited to specific conditions and relatively carefully selected groups, and that mortality is a relatively rare outcome which most trials will be underpowered to detect.

### Implications for practice and research

Our findings suggest that there may be a role for remote monitoring of vital signs for people with acute respiratory illness. We have not reviewed studies investigating the experience for the patient or caregiver of monitoring vital signs, but it is possible that this may help to provide a sense of autonomy or to improve self-care. It is also possible that self-monitoring may worsen anxiety or lead to increased consultations with community care services. While using remote monitoring, patients appreciate if the intervention provides security (clinical safety net), connection (link to their clinical team), empowerment (education on the disease and symptom management), user-friendliness (ease of access to services) and continuity of care provided. However, patients show concerns about additional burden (reluctance to learn something new, lack of trust in technology, avoiding additional out-of-pocket costs) and jeopardising interpersonal connections (fear of being lost in data, losing face-to-face contact) [27–29]. Future studies might consider a core outcome set to help reviews.

There are limited data on the implementation of remote monitoring as a complex intervention. In practice we do not think there is yet sufficient evidence for the widespread use of remote monitoring for people outside the COVID-19 pandemic, because there are limited data on the infrastructure needed to safely support the self-monitoring of vital signs. Most of the studies included in our review assessed patients with COPD. Thus, there is a need for further robust studies in a range of acute respiratory illness (*e.g.* asthma, pulmonary oedema and lower respiratory tract infection) to determine the true potential of remote monitoring in the acute phase of illness. Future studies should determine the most important outcomes for patients, clinicians and commissioners, should include a diverse range of patients and should report data on the patient experience of remote monitoring. Future trials should be explicit in defining the recruited population in terms of whether they are at risk of admission, eligible for early discharge or at risk of future exacerbations, and whether the care setting at recruitment is an emergency department, ward or community care setting. We recommend that future studies provide estimates of cost-effectiveness, sustainability and the impact in different socioeconomic–cultural settings, as well as using embedded qualitative methods to understand the most active and effective components of the intervention.

### Conclusion

The current evidence for remote monitoring suggests a beneficial effect on mortality. The impact on healthcare utilisation is particularly uncertain. We recommend further studies including cost-effectiveness analysis before widespread adoption in practice.

## Supplementary material

10.1183/23120541.00393-2022.Supp1 **Please note:** supplementary material is not edited by the Editorial Office, and is uploaded as it has been supplied by the author.
FIGURE S1 PRISMA 2009 Flow Diagram 00393-2022.supplementary_figure_S1
FIGURE S2 Sensitivity-analysis for mortality among all studies reporting outcome data (effect size represented as risk ratio) 00393-2022.supplementary_figure_S2
TABLE S1 Evidence to support author judgement for risk of bias in included randomised control trials 00393-2022.supplementary_table_S1

## References

[1] Greenhalgh T, Knight M, Inda-Kim M, et al. Remote management of covid-19 using home pulse oximetry and virtual ward support. BMJ 2021; 372: n677. doi:10.1136/bmj.n677 33766809

[2] O'Carroll O, MacCann R, O'Reilly A, et al. Remote monitoring of oxygen saturation in individuals with COVID-19 pneumonia. Eur Respir J 2020; 56: 2001492. doi:10.1183/13993003.01492-2020 32616588 PMC7331654

[3] Dhont S, Derom E, Van Braeckel E, et al. The pathophysiology of ‘happy’ hypoxemia in COVID-19. Respir Res 2020; 21: 198. doi:10.1186/s12931-020-01462-5 32723327 PMC7385717

[4] Couzin-Frankel J. The mystery of the pandemic's ‘happy hypoxia’. Science 2020; 368: 455–456. doi:10.1126/science.368.6490.455 32355007

[5] NHS England. Pulse Oximetry to Detect Early Deterioration of Patients with COVID-19 in Primary and Community Care Settings. 2020. www.england.nhs.uk/coronavirus/publication/pulse-oximetry-to-detect-early-deterioration-of-patients-with-covid-19-in-primary-and-community-care-settings/.

[6] Vindrola-Padros C, Singh KE, Sidhu MS, et al. Remote home monitoring (virtual wards) for confirmed or suspected COVID-19 patients: a rapid systematic review. EClinicalMedicine 2021; 37: 100965. doi:10.1016/j.eclinm.2021.100965 34179736 PMC8219406

[7] Sterne JAC, Savović J, Page MJ, et al. RoB 2: a revised tool for assessing risk of bias in randomised trials. BMJ 2019; 366: l4898. doi:10.1136/bmj.l4898 31462531

[8] Cochrane Training. Review Manager (RevMan). 2020. https://training.cochrane.org/online-learning/core-software/revman.

[9] Deeks J, Higgins J, Altman D. Analysing data and undertaking meta-analysis. *In*: Higgins J, Thomas J, Chandler J, et al., eds. Cochrane Handbook for Systematic Reviews of Interventions. 2021. www.training.cochrane.org/handbook.

[10] Koff PB, Min S-J, Freitag TJ, et al. Impact of proactive integrated care on chronic obstructive pulmonary disease. Chronic Obstr Pulm Dis 2021; 8: 100–116. 33238087 10.15326/jcopdf.2020.0139 PMC8047611

[11] Kessler R, Casan-Clara P, Koehler D, et al. COMET: a multicomponent home-based disease-management programme *versus* routine care in severe COPD. Eur Respir J 2018; 51: 1701612. 29326333 10.1183/13993003.01612-2017

[12] Vianello A, Fusello M, Gubian L, et al. Home telemonitoring for patients with acute exacerbation of chronic obstructive pulmonary disease: a randomized controlled trial. BMC Pulm Med 2016; 16: 157. doi:10.1186/s12890-016-0321-2 27876029 PMC5118881

[13] Chatwin M, Hawkins G, Panicchia L, et al. Randomised crossover trial of telemonitoring in chronic respiratory patients (TeleCRAFT trial). Thorax 2016; 71: 305–311. 26962013 10.1136/thoraxjnl-2015-207045 PMC4819626

[14] Jakobsen AS, Laursen LC, Rydahl-Hansen S, et al. Home-based telehealth hospitalization for exacerbation of chronic obstructive pulmonary disease: findings from “the virtual hospital” trial. Telemed JE Health 2015; 21: 364–373. doi:10.1089/tmj.2014.0098 PMC4432494 25654366

[15] Sorknaes AD, Bech M, Madsen H, et al. The effect of real-time teleconsultations between hospital-based nurses and patients with severe COPD discharged after an exacerbation. J Telemed Telecare 2013; 19: 466–474. doi:10.1177/1357633X13512067 24227799

[16] Chau JP-C, Lee DT-F, Yu DS-F, et al. A feasibility study to investigate the acceptability and potential effectiveness of a telecare service for older people with chronic obstructive pulmonary disease. Int J Med Inform 2012; 81: 674–682. doi:10.1016/j.ijmedinf.2012.06.003 22789911

[17] Kamei T. Information and communication technology for home care in the future. Jpn J Nurs Sci 2013; 10: 154–161. doi:10.1111/jjns.12039 24373438

[18] Vitacca M, Bianchi L, Guerra A, et al. Tele-assistance in chronic respiratory failure patients: a randomised clinical trial. Eur Respir J 2008; 33: 411–418. 18799512 10.1183/09031936.00005608

[19] Baroi S, McNamara RJ, McKenzie DK, et al. Advances in remote respiratory assessments for people with chronic obstructive pulmonary disease: a systematic review. Telemed JE Health 2018; 24: 415–424. doi:10.1089/tmj.2017.0160 29083268

[20] Kruse C, Pesek B, Anderson M, et al. Telemonitoring to manage chronic obstructive pulmonary disease: systematic literature review. JMIR Med Inform 2019; 7: e11496. doi:10.2196/11496 30892276 PMC6446156

[21] Polisena J, Tran K, Cimon K, et al. Home telehealth for chronic obstructive pulmonary disease: a systematic review and meta-analysis. J Telemed Telecare 2010; 16: 120–127. doi:10.1258/jtt.2009.090812 20197355

[22] Ram FSF, Wedzicha JA, Wright J, et al. Hospital at home for acute exacerbations of chronic obstructive pulmonary disease. Cochrane Database Syst Rev 2003; 4: CD003573. doi:10.1002/14651858.CD003573 14583984

[23] Janjua S, Carter D, Threapleton CJ, et al. Telehealth interventions: remote monitoring and consultations for people with chronic obstructive pulmonary disease (COPD). Cochrane Database Syst Rev 2021; 7: CD013196. doi:10.1002/14651858.CD013196.pub2 34693988 PMC8543678

[24] McLean S, Nurmatov U, Liu JLY, et al. Telehealthcare for chronic obstructive pulmonary disease: Cochrane review and meta-analysis. Br J Gen Pract 2012; 62: e739–e749. doi:10.3399/bjgp12X658269 23211177 PMC3481514

[25] Paré G, Jaana M, Sicotte C. Systematic review of home telemonitoring for chronic diseases: the evidence base. J Am Med Inform Assoc 2007; 14: 269–277. doi:10.1197/jamia.M2270 17329725 PMC2244878

[26] Taylor ML, Thomas EE, Snoswell CL, et al. Does remote patient monitoring reduce acute care use? A systematic review. BMJ Open 2021; 11: e040232. doi:10.1136/bmjopen-2020-040232 PMC7929874 33653740

[27] Daly B, Lauria TS, Holland JC, et al. Oncology patients’ perspectives on remote patient monitoring for COVID-19. JCO Oncol Pract 2021; 17: e1278–e1285. doi:10.1200/OP.21.00269 34085536 PMC8457795

[28] Walker RC, Tong A, Howard K, et al. Patient expectations and experiences of remote monitoring for chronic diseases: systematic review and thematic synthesis of qualitative studies. Int J Med Inform 2019; 124: 78–85. doi:10.1016/j.ijmedinf.2019.01.013 30784430

[29] Bouabida K, Malas K, Talbot A, et al. Remote patient monitoring program for COVID-19 patients following hospital discharge: a cross-sectional study. Front Digit Health 2021; 3: 721044. doi:10.3389/fdgth.2021.721044 34859244 PMC8630581

