# Supplementary figures and images for: Impact of remote vital sign monitoring on health outcomes in acute respiratory infection and exacerbation of chronic respiratory conditions: systematic review and meta-analysis
Source: ERJ Open Res. 2023 Apr 24;9(2):00393-2022. doi: 10.1183/23120541.00393-2022 (PMC10123516; doi:10.1183/23120541.00393-2022)

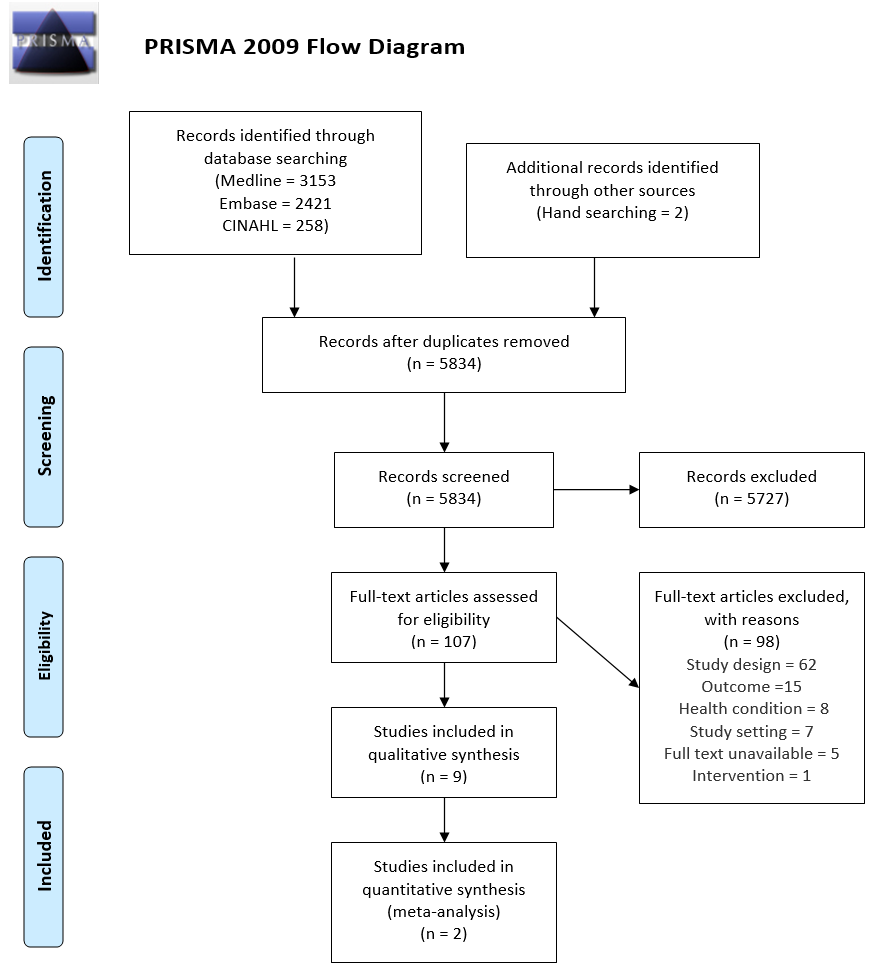

Supplement: Supplementary file 1 [file 00393-2022.supplementary_figure_S1.png]

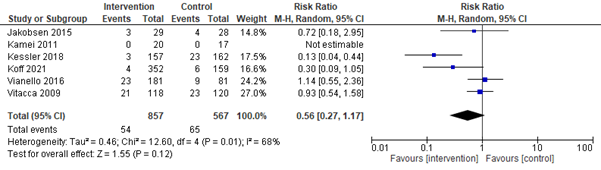

Supplement: Supplementary file 2 [file 00393-2022.supplementary_figure_S2.png]
