# Supplementary material for: Impact of remote vital sign monitoring on health outcomes in acute respiratory infection and exacerbation of chronic respiratory conditions: systematic review and meta-analysis
Source: ERJ Open Res. 2023 Apr 24;9(2):00393-2022. doi: 10.1183/23120541.00393-2022 (PMC10123516; doi:10.1183/23120541.00393-2022)
Supplement: Supplementary file 3 [file 00393-2022.supplementary_table_S1.pdf]

Supplementary Table 1 Evidence to support author judgement for risk of bias in included randomised control trials

Study	Cochrane Risk of Bias 2 Domain				
	Randomisation	Deviations from the intended intervention	Missing outcome data	Measurement of outcome	Reporting
**Koff 2021**	Quasi-randomized clinical trial Participants were allocated to experimental groups using a continuously rotating enrolment schedule with 4-day enrolment blocks (Proactive iCare group during the first 3 days of the block, and Usual Care group during the last day). No differences were found between groups for baseline parameters.	Each weekday, participants would participate in a Health Buddy session at home lasting during which they would measure their SpO2. Data were transferred to a database overnight for coordinators to view the next weekday.	A total of 511 participants were enrolled in the study, including 352 in the Proactive iCare treatment group and 159 in the Usual Care control group. A total of 122 participants did not complete the study.	Used intention-to-treat analysis. COPD education was given to each participant in intervention group, during weekday sessions with the Health Buddy and informally during phone calls with study coordinators. Participants in usual care group did not receive COPD education or advice but were advised to inform their health care provider if they had SpO2 ≤88%.	This trial was registered at www.clinicaltrials.gov (NCT01044927)
**Kessler 2018**	Patients were allocated to groups in a 1:1 fashion according to a pre-specified randomisation list generated before the study by a partial-minimisation computer algorithm under supervision of	For practical reasons, the study was open; neither the patients nor the investigators were blinded to the COPD management strategy.	A total of 345 patients were randomised to the DM group (172) or the UM group (173). In the DM and UM groups, respectively, 15 and 11 patients did not complete the initial 5-week run-in period.	Used intention-to-treat analysis. The control group received the usual or routine COPD care and patient follow-up practices used at each investigational centre. Site-specific usual management practices (e.g. centre-specific COPD	This trial was registered at www.clinicaltrials.gov (NCT01241526)

	the study sponsor. Patients were assigned a randomisation number by study staff at each centre in sequential numerical order through a telephone-based interactive voice response system. Randomisation was stratified by smoking status (current or former), need for respiratory assistance (none, or on LTOT and/or HMV), and centre. No differences were found between groups for baseline parameters.			educational booklets or programme information, if any) were collected at the beginning and end of the patient inclusion period.	
**Vianello 2016**	Randomisation was performed using a dedicated algorithm provided by PASS 2008 software that took into account patient's age and gender. Patients were randomised to the intervention or control groups using a 2:1	Using the TM equipment, patients transmitted their monitored Heart Rate and SpO2 values to the operator. If the measurement was outside of the patient's normal range, the operator. alerted the clinical staff.	The 334 eligible participants were randomly assigned: 230 were assigned to the TM group and 104 to the control one. Out of the 230 patients allocated to the study group, 19 did not actually participate in the	Used per-protocol analysis.	This trial was registered at www.clinicaltrials.gov (NCT01513980)

	allocation. Each participating center implemented randomization locally using the same methodology.<br>No differences were found between groups for baseline parameters.		study. At the end of the study, the data of 181 patients who had been randomized to the TM group and 81 control group, respectively, were available for analysis.		
**Chatwin 2016**	Patients were randomised to telemonitoring or delayed telemonitoring (control group), stratified for COPD or non-COPD diagnosis in blocks of five with results generated and made available from our statistics unit.<br>No differences were found between groups for baseline parameters.	Telemonitoring was carried out in the patient's home. The system requests daily responses to a questionnaire and responses were linked to the patient's television screen as an additional television channel that was accessed by hand-held remote. Each patient received education in using the monitoring devices until he or she and/or family/carer felt fully confident using it. Management was carried out by a combination of allied health professionals, predominantly clinical nurses in the hospital who	72 patients were randomised, 38 to telemonitoring first and 34 to control group first. 67 patients completed the first limb and at 12 months, analysis was completed in 61 patients.	Used intention-to-treat analysis.<br>Analysis was carried out blind to trial limb participation.	This trial was registered at www.clinicaltrials.gov (NCT02180919)

		were aware of group allocation.			
**Jakobsen 2015**	Patients were externally randomized 1:1 in fixed blocks of 4. The allocation sequence was hidden in sequentially numbered, sealed, opaque envelopes that were delivered to the hospitals in batches of 10. The sealed envelope was not opened by the patient until after the patient had signed a written consent form. The allocation concealment mechanism was monitored closely by the investigators to ensure that envelopes were never resealed and to ensure patients were entered correctly in the study no matter what allocation the envelope revealed. No differences were found between groups	It was not possible to blind patients or health professionals.	2 patients in the intervention group discontinued the intervention. 1 patient in the intervention group never received the allocated intervention. All patients in the control group received the allocated treatment. One patient in the control group was discontinued owing to suspicion of malignancy.	Used intention-to-treat analysis.<br>Statistician analyzing the data was blinded to allocation.	This trial was registered at www.clinicaltrials.gov (NCT01155856)

	for baseline parameters.				
**Sorknaes 2013**	Randomisation was performed centrally by a telephone voice response service from a computer-generated allocation sequence with a varying block size of 10 and 14. Participants were allocated to the two groups in a 1:1 ratio, and the randomisation was stratified by smoking status (current or ex-smoker versus never-smoker) and by trial site hospital 1 or hospital 2).<br>No differences were found between groups for baseline parameters.	Teleconsultations were conducted with patients in their homes and nurses at the hospital. The patient could take readings of the measurements on the telemedicine equipment, while the nurse collected the patient measurements electronically on a screen at the hospital.	266 patients were included in the present trial. Readmission could be determined in 261 patients at 4 weeks, in 257 patients at 8 weeks, in 253 at 12 weeks, and in 242 patients at 26 weeks.	Used intention-to-treat analysis.	The study was approved by the appropriate ethics committees.
**Chau 2012**	Patients were randomized to receive telecare or usual care following a simple randomization procedure (drawing a slip of paper with the group assignment	Participants in the intervention group were asked to monitor their SpO2 and pulse rate using the device and transmit the data to the online network platform. The community nurse who	53 patients underwent randomization and 30 were assigned to the intervention group and 23 to control group. 8 withdrew before the	Used intention-to-treat analysis.	Ethical approval was obtained from the Clinical Research Ethics Committee of the hospital.

	marked on the slip).<br>Pre- and post-test data were collected by research assistants who were not involved in the intervention.<br>No differences were found between groups for baseline parameters.	monitored the telecare system at home instructed the participants on the use of the device for self-monitoring during the home visit through demonstration and return demonstration.	intervention and 22 underwent it. 4 patients in the control group were lost to follow-up and 1 patient was hospitalised during the post-test data collection. 18 in control group were analysed.		
**Kamei 2011**	Language barrier<br>Research assistant randomly assigned the consented persons to the intervention group or the control group by the envelope method.<br>No differences were found between groups for baseline parameters.	Language barrier<br>The daily mental and physical condition of the patient was monitored at a remote location, and health / nursing guidance and mentoring were continuously provided.	Data of all patients randomised to study arms were analysed. No patient was lost during study period or follow up.	Used intention-to-treat analysis.	Language barrier<br>This study was carried out with the approval of the research ethics review committee of the affiliated and Cooperating institutions. However, the trial protocol was not registered.
**Vitacca 2009**	Using a set of computer-generated random numbers in 1:1 ratio patients were assigned to the treatment or control group.<br>No differences were found between groups for baseline parameters.	Patients recorded SpO2 using a finger pulse oximeter at home and the data was transferred to a TA nurse at the hospital, via a home telephone line.	All data reported in CONSORT flow diagram.	Used intention-to-treat analysis.	This trial was registered at www.clinicaltrials.gov (NCT00563745)
